# Evaluation of forelimb gait variation overground at a walk in sound and lame dogs using a combination of diagnostic techniques

**DOI:** 10.1186/s13028-024-00746-w

**Published:** 2024-06-20

**Authors:** Miriam Kjörk Granström, Lars Roepstorff, Kjerstin Pettersson, Ingrid Ljungvall, Maria Dimopoulou, Charlie Peck, Annika Bergström

**Affiliations:** 1 University Animal Hospital, Swedish University of Agricultural Sciences, Ultunaallén 5A, Uppsala, SE-75007 Sweden; 2 Department of Anatomy, Physiology and Biochemistry, Faculty of Veterinary Medicine, Swedish University of Agricultural Sciences, P.O. Box 7070, Uppsala, SE-75007 Sweden; 3 Department of Clinical Sciences, Faculty of Veterinary Medicine, Swedish University of Agricultural Sciences, P.O. Box 7070, Uppsala, SE-75007 Sweden

**Keywords:** Canine, Elbow, Gait analysis, Kinematic, Kinetic

## Abstract

**Background:**

Kinetic and kinematic gait analysis is increasingly practised as a part of lameness evaluation in dogs. The aim of this study was to examine the normal short- and long-term variation in forelimb gait in sound control dogs (CD) at a walk using seven selected variables of objective kinetic and kinematic gait analyses. Also, to compare the findings in CD to a group of forelimb lame dogs with elbow osteoarthritis (OAD). An additional aim was to test a kinetic based graphic method for lameness detection; symmetry squares (SS). A prospective longitudinal study was carried out on client owned CD and OAD. Clinical and orthopaedic evaluations were performed to ensure soundness and detect and grade lameness. Seven kinetic and kinematic variables and SS were tested for lameness evaluation. The CD were divided into two subgroups, CD1 and CD2, and examined twice: CD1 with two months interval and CD2 with 3–4 h interval. The OAD group was evaluated once and compared to the CD groups’ first examination.

**Results:**

Thirteen CD and 19 OAD were included. For CD1 and CD2, there were no significant differences in any examined variable between examination occasions. Total peak force/impulse symmetry and fore-hind peak force/impulse symmetry differed significantly between OAD and CD. Symmetry squares had a 74% agreement to subjective orthopaedic evaluations.

**Conclusions:**

In CD, no difference in the examined variables was seen between examination occasions. Four out of seven objective variables differed significantly between CD and OAD. The graphic SS method might have diagnostic potential for lameness detection, making it possible to detect a shift from lame to non-lame limbs. Potentially, this might be especially helpful in bilaterally lame dogs, which often represent a clinical challenge in lameness evaluation.

**Supplementary Information:**

The online version contains supplementary material available at 10.1186/s13028-024-00746-w.

## Background

Consistent and reliable assessment of lameness in dogs remains a clinical challenge, especially in cases of subtle lameness [1, 2]. A considerable variability in gait can occur even in clinically sound dogs [3], which can be assumed to complicate lameness assessment in the presence of orthopaedic diseases. Knowledge of the normal variation span in frequently used gait parameters in non-lame dogs is crucial to define and identify significant changes. Understanding normal variation is necessary for clinical research as well as for appropriate assessment of each patient’s improvement or development of an orthopaedic ailment.

*Visual assessment* of gait is commonly performed in clinical practice to identify lameness. However, unless lameness is severe, visual assessment has been described to show substantial interobserver variation and poor agreement with objective kinetic analysis, as previously stated [1, 4].

*Kinetic gait analysis* is the study of forces generated during locomotion. Ground reaction forces (GRFs) can be measured using a force plate [1]. Peak vertical force (PF) and vertical impulse (I) are frequently used force plate variables and may be considered gold standard for the evaluation of weight bearing lameness in dogs [1, 4–10].

*Kinematic analysis* of gait is the study of motion and quantifies variables that describe the location and motions of body segments in space. Markers are placed on predetermined anatomic landmarks, with reflective markers being the most commonly used landmarks for three-dimensional (3D) kinematics. Cameras record the markers’ locations during locomotion on a treadmill or over ground. Specialized software [11] supplies 3D-coordinates of markers over time.

There are different inherent limitations with each of the methods mentioned above for lameness/gait asymmetry evaluation. Combined tests for evaluation of lameness are therefore reported and clinically used in human orthopaedics [12–14], which produces more accurate results than each method separately [11]. Recently, combined tests for stifle function in dogs, including subjective and objective variables, have been suggested [15, 16].

The first aim of this study was to examine the normal variation in several kinetic, kinematic and subjective gait variables in clinically sound control dogs (CD) at a walk; then compare these results to similar variables in forelimb lame dogs with elbow OA (OAD). Secondly, we aimed to investigate the normal variability in load distribution in CD over time, with measurements repeated the same or after two months. We also tested a kinetic-based graphic method, “Symmetry Squares” (SS), depicting the load of all four legs. An additional aim was to compare this graphic method to orthopaedic lameness evaluation in a pilot test of its diagnostic value and potential as a future clinically applicable tool to aid in detection of lameness.

## Methods

### Dogs

#### Inclusion criteria

All dogs eligible to participate in the study were non chondrodystrophic individuals weighing 20–40 kg with an estimated stride length that allowed measurement of one stance phase on the force plate. This inclusion criterion maximized the chances of hitting the force plate with one paw at a time with the least possible attempts. Dogs should have reached their estimated full height, and their general health was not to be affected by a condition potentially influencing the orthopaedic assessment. Dogs with elbow osteoarthritis (OAD group) and control dogs (CD group) without signs of lameness according to clinical and subjective orthopaedic evaluations, were included and divided in to two separate groups.

The OAD had to have uni- or bilateral forelimb lameness and elbow osteoarthritis confirmed by radiography or computed tomography evaluated by a board-certified radiologist (ECVDI). Any ongoing pain medication was recorded. The OAD were also part of a parallel study [17].

The study was performed at the University Animal Hospital, Uppsala, Sweden, and was ethically approved by the Uppsala animal ethics committee (C102/15). All dog owners provided signed informed consent prior to enrolment in the study.

#### Exclusion criteria

For CD, exclusion criteria were history of lameness, presentation with lameness at a walk or trot on visual gait assessment and signs of joint disease during orthopaedic examinations. Additionally, ongoing medication with non-steroidal anti-inflammatory drugs, corticosteroids or other drugs that could potentially mask pain responses was a criterion for exclusion.

The OAD were excluded if clinical signs of lameness, originating from other than the elbow joints on visual assessment of gait and orthopaedic examinations, were found; including pain on palpation, abnormal range of motion, thickened joint capsules, or other musculoskeletal abnormalities unrelated to the elbow joints.

#### Subgroups and number of examinations

The CD group was divided into two subgroups. Group CD1 was examined twice with a two month interval. Group CD2 was examined twice during the same day, with at least three hours of rest and a short leash walk between the examinations. Data from the OAD group were collected once for the current study.

#### Clinical evaluations

All dogs were independently examined by two experienced clinicians: one board certified surgeon (AB) and a veterinary certified physiotherapist (KP). At first, gait was visually assessed on a concrete walkway outside the University Animal Hospital, at both a walk and a trot. During gait assessment, dogs were handled by their owners or by one of the authors (MK). Lameness was subjectively rated by both examiners using a numerical rating score (NRS) of increasing lameness graded 0–5 [18, 19].

Gait assessment was followed by clinical and orthopaedic examinations (AB) and a physiotherapist evaluation including orthopaedic examination as well as muscle circumference measurements of the brachium, thigh and crus and measurement of elbow passive range of motion (PROM) by goniometry (KP) [20]. Goniometry and muscle measurements for each dog were compared between examinations using the same reference points [21]. Orthopaedic palpable pain and PROM was assessed and graded as none (0), mild (1), moderate (2) or severe (3).

After discussing all of the subjective individual variables (AB and KP), a consensus score was set for each dog; who accordingly was put into one of three categories: normal, mildly, or moderately to severely affected (Table 1). The consensus score was based on the following clinical variables: lameness, joint pain and PROM. The purpose of using a consensus score in the study was to amalgamate and reduce the number of variables, while simultaneously evaluating an average of the clinical data for any deviations.

**Table 1:** Summary of dog characteristics, orthopaedic evaluations, and kinetic- and kinematic objective measurements in control dogs

	Control dogs (CD1 + CD2)	Dogs with osteoarthritis (OAD)	*P*-value
Number	13	19	NA
Age	5.2 ± 3.1	4.2 ± 2.9	0.27
Sex (female/male)	8/5	10/9	0.62
Body weight	28.8 ± 6.6	35 ± 8.9	0.06
BCS, U/N/O • U:1–3 underweight • N:4–5 normal weight • ≥6 overweight	0/6/7	0/5/14	NA
***Subjective orthopaedic evaluations***			
Lameness score trot (0/1/2/3/4/5)	Dx: 13/0/0/0/0/0 Sin: 13/0/0/0/0/0	Dx: 2/3/5/2/5/0 Sin: 2/4/3/4/3/0	NA
Consensus score ^I^ (normal/mild/moderate&severe)	Dx: 13/0/0 Sin: 13/0/0	Dx: 3/8/8 Sin: 4/9/6	NA
Measured ROM elbow (degrees)	Dx: 124.9 ± 3.1 Sin: 124.9 ± 3.2	Dx 115.6 ± 11.3 Sin 120.2 ± 7.3	Dx: 0.006 Sin: 0.09
Measured muscle mass (cm)	Dx: 27.2 ± 2.0 Sin: 27.5 ± 1.9	Dx: 28.7 ± 2.5 Sin: 29.5 ± 2.9	DX: 0.12 Sin: 0.04
Asymmetry Symmetry Squares (Yes/No)	0/13	14/5	NA
***Objective kinetic and kinematic evaluations***			
Peak Force	Dx FL: 6.68 ± 0.43 Sin FL: 6.75 ± 0.52 Dx HL: 3.95 ± 0.43 Sin HL: 4.03 ± 0.52	Dx FL: 6.58 ± 0.69 Sin FL: 6.46 ± 0.86 Dx HL: 4,48 ± 0,53 Sin HL: 4.55 ± 0.46	0.5267 0.4315 0.0023* 0.0020*
Impulse	Dx FL: 2.60 ± 0.28 Sin FL: 2.68 ± 0.30 Dx HL: 1.47 ± 0.14 Sin HL: 1.52 ± 0.18	Dx FL: 2.63 ± 0.38 Sin FL: 2.57 ± 0.48 Dx HL: 1.62 ± 0.25 Sin HL: 1.58 ± 0.22	0.9847 0.6315 0.1296 0.3279
Total PF Symmetry	-1.9 ± 1.6	-7.1 ± 4.9	0.0001*
Fore-Hind PF Symmetry	-0.7 ± 1.6	-4.34 ± 3.4	0.001*
Fore PF Symmetry	-0.3 ± 1.1	0.5 ± 5.4	0.77
Total Impulse Symmetry	-2.2 ± 1.7	-5.2 ± 3.7	0.002*
Fore-Hind Impulse Symmetry	0.3 ± 1.4	-1.7 ± 2.7	0.003*
Fore Impulse Symmetry	-0.5 ± 1.8	0.6 ± 5.6	0.77
Kinematic ROM elbow (degrees)	Dx: 55.4 ± 5.1 Sin: 55.4 ± 5.8	Dx: 57.8 ± 6.1 Sin: 58.2 ± 7.4	DX: 0.16 Sin: 0.16

Control dogs (CD) including dogs examined twice with two months interval (CD1) and twice the same day (CD2), and lame dogs with elbow osteoarthritis (OAD). Orthopaedic evaluation data derived from the first day of examination/ day of inclusion in the study. Values are reported as mean, with standard deviations (±) when applicable. Group-wise comparisons were performed using the Wilcoxon rank sum test and a value of < 0.05 was considered significant. P-value not available is stated as (NA).

I. Consensus subjective overall score of combined orthopaedic evaluations (veterinarian and physiotherapist), including lameness score at a walk and trot, pain score and passive ROM. Asymmetry is based on evaluation of blinded observers

BCS = body condition score, ROM = range of motion, PF = peak force Dx = dexter Sin = sinister FR = forelimb, HL = hindlimb

### Kinetic and kinematic evaluations

#### Data collection

Overground kinetic and kinematic measurements and registrations were performed twice for the CD1 and CD2 groups and once for the OAD-group. High-speed cameras and a submerged 40 × 60 cm piezoelectric force plate (Kistler model 9286 B) were used. For details about the setup, see Additional file 1. The following variables were examined: Total Peak Force (PF) Symmetry (Sym), Fore-Hind PF Sym, Fore PF Sym, Total Impulse(I) Sym, Fore-Hind I Sym, Fore I Sym and Kinematic Range of Motion (ROM) elbow. The dogs were led by their owners or one of the authors (AB) on a leash with no tension applied. Trials were considered valid for further analysis when the dog walked at an even pace, straight forward without turning its head and with one paw at a time striking the FP close to the centre of the plate. Two observers evaluated the trials in real time, and collected data were also visually double-checked. Trials were repeated until data from six valid trials were obtained for each limb.

#### Symmetry squares (SS)

**Fig. 1:**
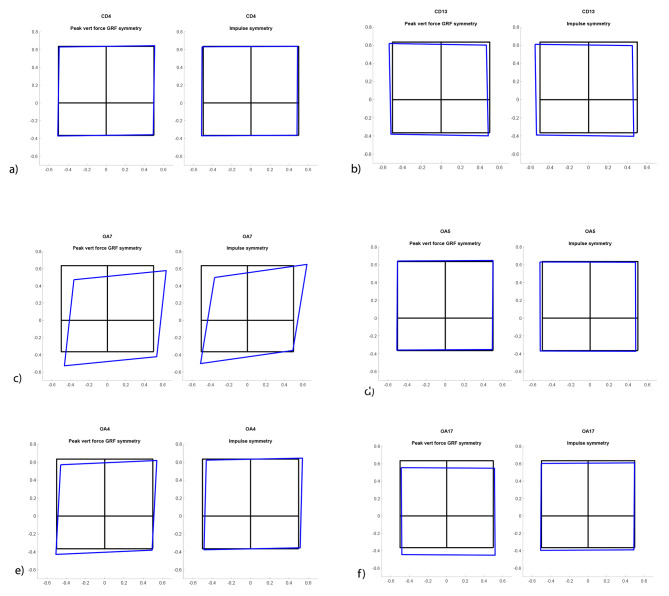
Symmetry squares (SS) from six different dogs illustrating load distribution between all four legs. The black square, with each corner representing a limb, illustrates the mean of control dogs. Each ¼ corner of the square represents a leg as seen from a dorsal view. Peak Vertical Force SS to the left and Vertical Impulse (I) SS to the right. **a**) CD4, very well fitting the reference square, the figure also includes an explanation of how the figures should be interpreted, with one limb in each corner of the square. **b**) CD13, deviations from reference square representing normal variation, **c**) OAD7, left forelimb lameness clearly seen as transferring of weight from the painful limb – the blue square shifts to the right. More negative values correspond to larger forces transferred to the hind limbs. **d**) OAD5, bilateral forelimb lameness, not possible to detect from the squares **e**) OAD4, bilateral forelimb lameness more severe on the left side, seen as transferring of weight to the hindlimbs and primarily to the right **f**) OAD17, symmetric bilateral forelimb lameness, seen as transferring of weight to the hindlimbs. CD = control dogs, OAD = dogs with elbow osteoarthritis, SS = symmetry squares

Based on the results from the CDs´ PF and I- measurements, a symmetry graph was constructed depicting the load distribution between all four legs for each dog (Fig. 1). Kinetic data from each dogs’ examination event were assigned a defined colour and projected together with a reference black square. Any deviation from the black square represents a shift of load. The results of the CD-group were compared to the results of the OAD- group in order to assess visual differences in gait patterns. For details, see Additional file 2. After a brief introduction to the method, three observers, blinded to all information regarding the dogs identities and lameness status, were asked to determine whether a dog was lame or not, based only on information from the 29 SS-graphs. All observers were experienced in performing orthopaedic examinations. In dogs assessed to be lame, the observers were to decide which leg(s) that was/were affected and if the dogs walked symmetrically or not. These results were thereafter compared to the orthopaedic consensus score. Two SS-graphs were constructed for each dog based on PF and I- measurements, respectively. The paired graphs were reviewed independently by the three observers. Anonymized and randomized SS graphs from the first examination for all CD and OAD- dogs were sorted into one of the following categories: normal, lame-bilateral, lame-left forelimb, lame-right forelimb, lame-bilateral left dominant, lame-bilateral right dominant. Thereafter, the panel members reviewed and discussed the cases they disagreed on to identify the reasons for disagreement and to evaluate if consensus could be reached. From these results, asymmetry deteced from the SS graph is presented (Table 1).

#### Data analysis

The details regarding data analysis and capturing of kinetic data via a Kistler force plate are found in Additional file 3. The motion capture software Qualisys Track Manager® (version 2.12) automatically calculated and reconstructed the three-dimensional coordinates of each reflective marker. Each marker was manually identified, labelled, and validated for correct tracking. For further analysis, marker data were exported to Matlab (R2013B) using custom made scripts.

The following temporospatial and kinetic variables were measured: stance time, peak vertical force (PF), vertical impulse (I) and elbow range of motion (ROM). Gait velocity was measured in m/s. Kinetic variables were normalized for body weight. Speed was normalized to mean speed at each session for each dog (Additional file 1, “Collection of objective data”). To further analyse the relative distribution of forces, the following symmetry variables were calculated for PF and I for each dog: total symmetry between all four legs, forelimb-hindlimb PF symmetry and left-right forelimb symmetry.

### Statistical analysis

Statistical analyses were performed using a commercially available software program ^I^. Data was analyzed using descriptive as well as inferential statistics. Continuous variables were presented as mean and standard deviation (SD). The chi-squared test was used to test for differences in proportions in sex between CD and OAD. Continuous data listed in Table 1 were analyzed between the groups (CD and OAD) using the non-parametric Wilcoxon rank sum test. A value of *P* < 0.05 was considered significant.

For the outcome of biomechanical and measured orthopaedic variables listed in Table 2, differences between time-points of examinations (two months apart for the CD1 group of dogs and three-four hours apart for the CD2 group of dogs, respectively) were investigated. A mixed linear model was used, including dog identity as a random variable, and time-point of examination. For the distributions of model residuals, normality was ensured by visual inspection.

**Table 2:** Summary of results in non-lame dogs (CD) examined twice

	CD1 1st (*n* = 8)	CD1 2 months (*n* = 8)	*P* -value	CD2 1st (*n* = 5)	CD2 2nd (*n* = 5)	*P*- value
***Subjective evaluations***						
Measured ROM elbow (degrees)	Dx: 125.6 ± 2.9 Sin: 125.4 ± 1.3	Dx: 124.5 ± 0.3 Sin:124.9 ± 0.2	0.25 0.67	Dx: 123.6 ± 3.3 Sin:124.0 ± 2.6	NA	NA
Measured muscle mass (cm)	Dx: 26.6 ± 2.0 Sin: 27.0 ± 2.0	Dx: 26.8 ± 2.0 Sin: 27.2 ± 2.0	0.74 0.76	Dx: 28 ± 1.7 Sin: 28.1 ± 1.8	NA	NA
***Objective evaluations***						
Total PF Symmetry	-1.6 ± 1.6	-2.3 ± 1.6	0.31	-2.2 ± 0.7	-1.8 ± 0.4	0.58
ForeHind PF Symmetry	-1.0 ± 1.5	-0.8 ± 1.7	0.76	-0.4 ± 1.7	0.1 ± 1.6	0.79
Fore PF Symmetry	0.2 ± 0.5	0.1 ± 1.1	0.85	-1.1 ± 1.4	0.3 ± 1.1	0.40
Total Impulse Symmetry	-1.6 ± 0.6	-1.9 ± 0.8	0.41	-3.1 ± 1.11	-2.8 ± 1.7	0.47
ForeHind Impulse Symmetry	0.6 ± 1.0	0.4 ± 1.3	0.80	-0.1 ± 2.0	0.6 ± 1.7	0.29
Fore Impulse Symmetry	-0.1 ± 0.9	0.2 ± 1.0	0.58	-1.3 ± 2.6	-0.4 ± 2.1	0.21
Kinematic ROM elbow (degrees)	Dx: 54.4 ± 5.8 Sin: 56.8 ± 6.5	Dx: 53.4 ± 7.4 Sin: 50.9 ± 8.3	0.55 0.09	Dx: 56.9 ± 39.9 Sin: 53.1 ± 4.0	Dx: 55.2 ± 4.3 Sin:51.7 ± 2.4	0.51 0.27

CD1 was examined two months apart and CD2 the same day, two to four hours apart. Values are reported as mean, with standard deviations (±) when applicable. There was no significant difference in any value between the two examinations

ROM = range of motion, PF = peak force, CD = control dogs

## Results

Thirteen dogs in the CD group and 19 dogs in the OAD group were included in the study. Four dogs were excluded due to lameness and/or pain not related to the elbow joints. There were no significant differences in age, body weight or sex between groups. Breeds represented in the CD group were Belgian malinois (*n* = 1), German shepherd (*n* = 2) German wirehaired pointer (*n* = 1), Labrador retriever (*n* = 5), Mixed breed (*n* = 1), Rottweiler (*n* = 2), and Smooth collie (*n* = 1). Breeds represented in the OAD group were Belgian malinois (*n* = 1), Bernese mountain dog (*n* = 2), Bullmastiff (*n* = 1), German shepherd (*n* = 3), German spaniel (*n* = 1), Labrador retriever (*n* = 7), Leonberger (*n* = 1), Mixed breed (*n* = 2) and Rottweiler (*n* = 1).

The clinical orthopaedic, as well as kinetic and kinematic measurements and comparisons between groups are presented in Table 1.

There were no significant differences between first and second examination in any tested parameter; neither when examination was repeated after a few hours (CD2) nor after two months (CD1) (Table 2).

**Fig. 2:**
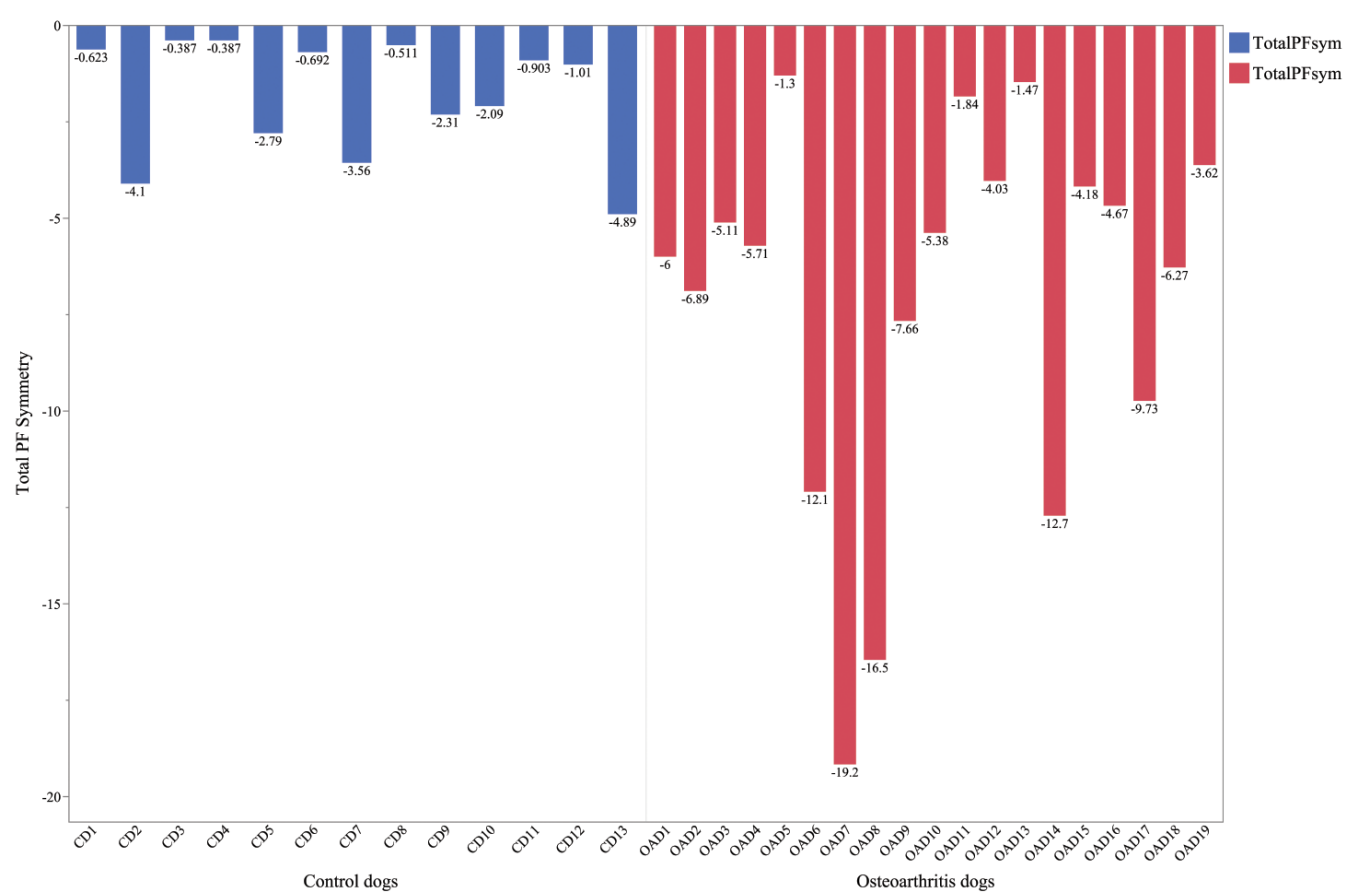
The median value of the Total PF Symmetry for each dog. Control dogs (CD) CD (blue) to the left and dogs with elbow osteoarthritis (OAD) (red) to the right. Overall, Total PF Symmetry was lower and showed a greater variation span for OAD compared to CD. However, there was a noticeable area of overlapping between groups. PF = peak force, CD = control dogs, OAD = dogs with elbow osteoarthritis

The variables Total PF Sym, Fore-Hind PF Sym, Total I Sym, Fore-Hind I Sym and measured ROM right elbow differed significantly between the CD and OAD groups. The asymmetry and diverging Total PF, as well as the overlap in Total PF between CD and OAD, is presented in Fig. 2. Blinded evaluation of SS initially resulted in 70.4% inter-observer agreement (Table 3). After consensus discussion, a 100% inter-observer agreement was reached. Figure 1 shows a selection of SS illustrating variation in load distribution in non-lame CD and lame OAD. When comparing the observer’s consensus SS evaluation to the orthopaedic consensus evaluation, agreement was found on 13/19 (68%) of the OAD and 7/8 of the CD (87.5%); in total 74% agreement. Non-agreement was found in 2/13 unilaterally lame dogs and 3/6 bilaterally lame dogs.

**Table 3:** Results from three blinded observers individually evaluating symmetry squares

Level of agreement	No. of cases
Full agreement, 3/3 observers	19
Three different opinions	0
Full agreement on the most lame leg, 2/3 also agreed on bilateral lameness	5
2/3 observers agreed on lame leg/ normal variation	3

Nineteen dogs with elbow osteoarthritis (OAD), bi-or unilaterally forelimb lame, and eight non lame control dogs (CD)

## Discussion

The results from this study show that Total PF and Total I as well as Fore-Hind PF- and Fore-Hind I symmetry differed significantly between CD and OAD. In CD, these variables did not change between repeated examinations within a period of two months. Also, a kinetic-based graphic method (SS) was tested and found to have 74% agreement with subjective orthopaedic evaluation.

Total PF and Total I as well as Fore-Hind PF and Fore-Hind I- symmetry all differed significantly between OAD and CD in the current study. In accordance with these results, several earlier studies have identified PF and I as the parameters best correlating with limb function [9, 10, 22]. There are contradicting results in the literature regarding consistence over time of Total PF and Total I. Some observers report PF to be consistent over time at a trot using a force plate and a pressure mat [23, 24]. In contrast, inter-day and -week differences have been reported in PF of the forelimbs of healthy dogs at a trot, which potentially could be attributed to habituation [25]. In the present study, only non-significant variations between first and second evaluation of CD were found in all tested parameters and for both time intervals when examinations were performed under similar conditions. Notably, although lame dogs were more asymmetric and showed more diverging results, an overlap between CD and OAD was seen. Lame dogs can have a Total PF equal to normal dogs [10, 26]. This emphasizes the complexity in lameness assessment and may imply that objective methods should be seen as an important complement when evaluating lameness, rather than an absolute truth - as there are no specific thresholds to distinguish lame from sound. A multivariable approach to lameness has previously been suggested as there is no existing consensus for a single specific diagnostic test to evaluate limb function in dogs [1, 10, 26]. To the authors’ knowledge, there exists no evaluation method combining objective and subjective measurements for elbow assessment in dogs to this date. However, it is likely that a combination of multiple diagnostic methods might delineate the most accurate picture of the lame dog.

Symmetry squares were based on PF- and I values and aimed to present “a clinically applicable and intuitive to understand diagnostic tool” that could potentially facilitate gait assessment in dogs in terms of weight distribution. A 74% agreement of SS was acquired with the orthopaedic consensus evaluation, which suggests the method might be a helpful addition in the clinical setting, possibly more so for long-term follow up of a single patient. Further prospective studies are warranted to evaluate the use of SS when assessing lameness in a single patient over time. For 19/27 dogs, all three observers separately, preceded by just a brief familiarization with the method, assessed SS identically. It is important that presentation of data is made intuitively easy to understand, which was the reason for why we at first hand wanted to test the ability of clinicians to interpret data based on a very brief introduction instead of a detailed walk through. The cases for which interobserver agreement was low when evaluating SS were either cases with bilateral forelimb lameness or control dogs with small deviations from normal gait symmetry, according to the other variables tested. With increasing experience of SS as a lameness evaluation tool, the sensitivity and specificity of the method may potentially improve. Symmetry squares seem to be simple to understand for clinicians working with orthopaedics and might be an additional evaluation method for detecting lameness. Total PF and Total I are also possible to obtain from a pressure mat, which potentially can make SS easier and cheaper to apply clinically compared to the force plate [23, 27–29]. Previously, studies have been done on vertical force distribution in forelimb lame vs healthy dogs [30]. It is likely that these kinds of visual techniques will be used more in the future to help understand how the load is distributed in lame dogs.

In the OAD group, a reduced kinematic ROM due to pain from OA elbows could have been expected, which we did not find. Our results are similar to other studies [31], who also compared normal and OA diseased elbows. As seen in Table 1, there was a greater variability in measured ROM in the OAD-group compared to the uniform CD-group, while there was no significant difference between the groups regarding kinematic ROM. Kinematic ROM at a walk is not directly comparable with measured ROM performed in lateral recumbency with the joint in maximal flexion/extension. In 1995, a study [32] tested repeatability of kinematic measures with three-week intervals in normal Greyhounds, indicating negligible variance.

Objective gait analysis was performed at a walk as some patients were not able to trot long enough for valid hits on the force plate. As [33] argues, excluding these individuals would pose a risk for selection bias towards less lame dogs. Differences between e.g. a control versus a treatment group might then appear smaller. Therefore, results from normal variation measurements at a walk could possibly be more clinically applicable.

There are several limitations to this study. There was a relatively small number of included dogs. Skin motion artifacts are a well-known concern in kinematic gait analysis [34, 35]. We tried to minimize artifacts by standardizing standing positions during marker placement [36], clipping fur and having the same person apply all markers. We used a single FP, which restricted the calculations to non-consecutive steps. The importance of this limitation is debatable [25, 37].

Orthopaedic evaluations are subjective with a risk of bias. By integrating a range of variables and meticulously calculating a consensus score, we attempted to reduce the method’s shortcomings. Factors such as bilateral lameness and individual dog reactions may still influence the outcome. Bilateral lameness is common in dogs and is considered more difficult to evaluate subjectively as well as objectively. There is a risk that bilateral lameness - especially if symmetrical, is identified as normal variation. Including bilaterally lame dogs can be regarded as a significant limitation to the study. However, a significantly bigger peak force shift from fore- to hind limbs was seen in OAD compared to in CD. SS makes this weight shift from lame forelimbs to hind limbs visible, which facilitates detection of forelimb lameness. Accordingly, SS is a potentially helpful tool for visualizing this load shift.

The variation potentially attributed to the dog handler in kinetic studies varies between 0 and 7% for the GRFs evaluated [38] and is considered to be of little concern. Therefore, this is regarded as an insignificant source of error in the present study.

Velocity was not strictly regulated. Researchers have previously concluded that a wider velocity range can be used with little or no effect on GRFs in sound dogs, at least at the trot, suggesting negligible interference from this factor [39].

## Conclusions

Total PF and Total I, Fore-Hind PF and Fore-Hind I symmetry all differed significantly between OAD and CD. No difference was seen between examinations repeated the same day or after two months in the CD groups. Importantly, for all tested parameters there was an overlap between CD and OAD dogs. Symmetry squares appears to have potential as a complementary objective tool for lameness evaluation, making it possible to detect a shift from lame to non-lame limbs. Potentially, this might be especially helpful in bilaterally lame dogs, which often represent a clinical challenge in lameness evaluation. However, the method needs further evaluation in future studies.

### Electronic supplementary material

Below is the link to the electronic supplementary material.

Supplementary Material 1

## Data Availability

The datasets used and analyzed during the current study are available from the corresponding author on reasonable request.

## Declarations

### Prior publication

Data have not been published previously.

### Endnotes

Footnote I: JMP 16 Pro, Cary, NC USA.
