# Supplementary material for: Evaluation of forelimb gait variation overground at a walk in sound and lame dogs using a combination of diagnostic techniques
Source: Acta Vet Scand. 2024 Jun 20;66:25. doi: 10.1186/s13028-024-00746-w (PMC11191351; doi:10.1186/s13028-024-00746-w)
Supplement: Supplementary file 1 — Supplementary Material 1 [file 13028_2024_746_MOESM1_ESM.docx]

**ADDITIONAL FILES**

**Additional file 1.** Collection of objective data

The dogs were led by their owners or one of the authors at a walk at preferred walking velocity along a 5 m long walkway. In the middle of the walkway a 40x60 cm force plate (FP) were centrally positioned (*Kistler model 9286 B*), for collection of kinetic data. To ensure that the dogs would not notice the FP and due to that alter their locomotion pattern, the FP was submerged under and level with the floor and covered with the same non- slippery carpet. Prior to each data capturing, dogs were familiarized with walking along the walkway passing over the force plate, until they were clearly undistracted by the equipment and walked at an even pace. Exact positions of force plate corners were defined in the calibrated kinematic system thus enabling extra control of paw placement on the force plate. Any paw hits at the boarder of the force plate were discarded.

Force data were low pass filtered forward and backward with a Butterworth low pass filter with a cut off frequency of 100Hz. Any offset was removed by taking an average of 400 samples at the beginning of recording, which is clearly before any paw has hit the force plate and subtracting from the whole data series.

Force data was normalized to body weight. Kinetic data is sensitive to speed so correlation, within each session and for each dog for all valid force plate hits, between speed versus peak force and impulse were calculated and if there was a significant correlation peak force and impulse were normalized to the mean speed.

Optical motion capture was used to collect the kinematic data. Spherical reflective markers (12 mm in diameter) were glued (Karlssons klister™) to the skin overlying bony landmarks, as shown in Figure 1. Markers were consistently placed by the same person (ZZ). The overlying fur was shaved prior to marker placement, if needed. The kinematic data was recorded with eight infra-red three- dimensional cameras (*Qualisys Oqus®700+*) capturing at a frame rate of 500 Hz. The cameras were evenly spaced in semicircles, four cameras on each side of the walkway.


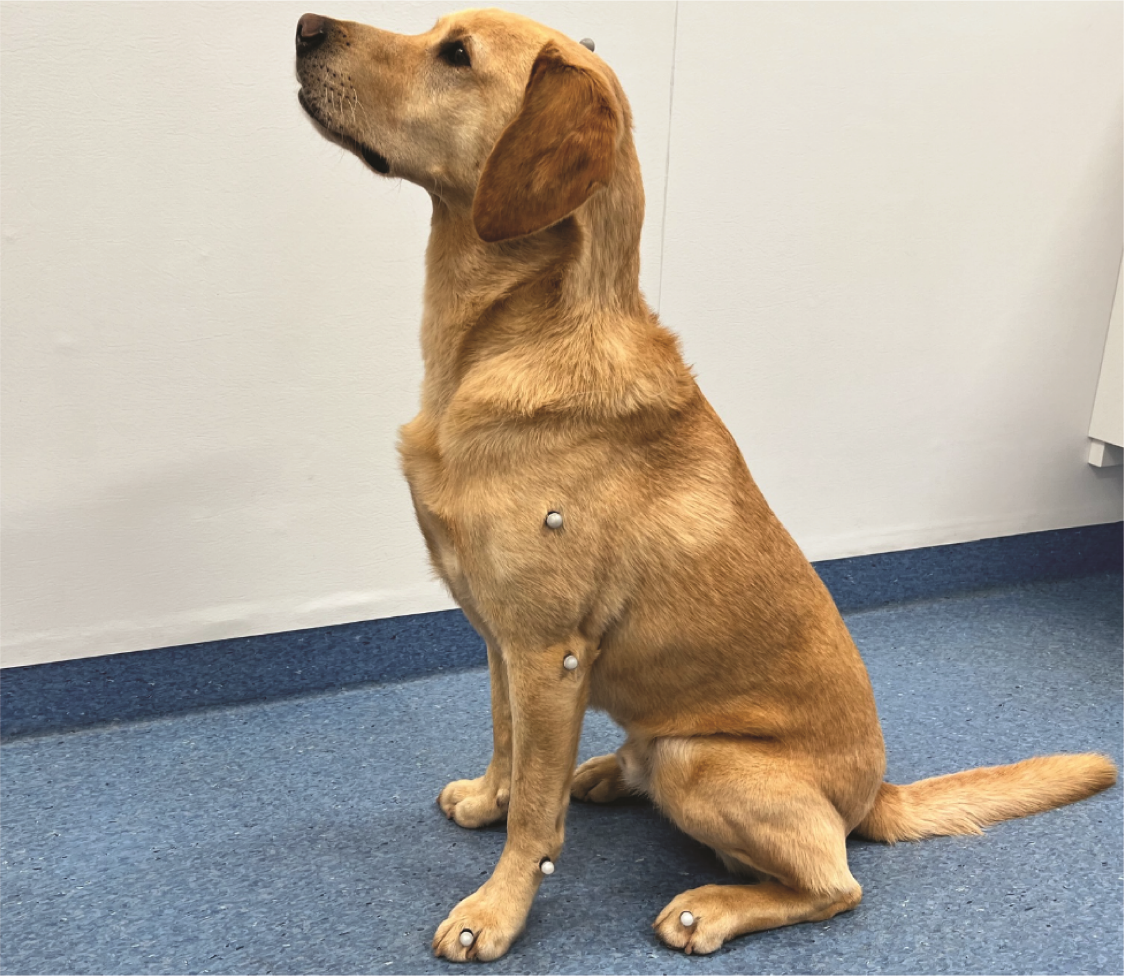


Figure 1 Additional file part 1. Four markers were glued to each forelimb; at the proximal interphalangeal joint on the fourth digit, the styloid process of ulna, the lateral epicondyle of humerus and the acromion. Also, one marker was placed on the proximal interphalangeal joint on the fourth digit on each hindpaw and one on the external occipital protuberance*.*

**Additional file 2.** Symmetry squares

Each ¼ corner of the square represents a leg, seen from a dorsal view. The horizontal line that divides the upper and lower squares is offset downwards, representing the normal weight distribution between fore- and hind limbs. “Normal” in this case is based on the CD- group; the mean weight distribution was 63.5% and 36.5% for fore- and hind limbs respectively. A square represents for each leg the relative peak vertical force (PF) or vertical impulse (I). A totally symmetric non-lame dog would fit the black “normal reference square” with perfectly straight angles. Kinetic data from each dog´s examination event was assigned with a defined color and projected on the reference square. The colored square fits on, or deviates from, the reference square according to how much load the dog relatively puts on each leg respectively.

For example: If a dog would have a left-right forelimb peak force distribution of 48/52 % the upper left and right corner of the square would be skewed 0.02 to the right. The CD-group were compared to the OAD- group to look for visual differences in gait patterns.

**Additional file 3**. Data processing

First toe on and toe off was defined from vertical ground reaction force by using the function “findchangepts” with settings 'Statistic', 'linear', 'MinThreshold'. The procedure was performed in two steps. First the function was used on cumulative sum of vertical ground reaction force, which creates very clear infliction points. These infliction points produce a very consistent and repeatable approximate definition of toe on and toe off. In the second step the same function with the same settings was used on the vertical ground reaction force to fine tune the toe on and toe off definition from the first step. The whole procedure was automated by iterating the threshold so the function only found infliction points that corresponded to toe and on toe off in each file respectively. While the procedure was running it was scrutinized manually to ensure no erroneous definitions were recorded.

Both kinetic and kinematic data was then time normalized to 100% of stance time (toe on to toe off).

A representation of protraction/retraction angle was defined as the 3-point angle between acromion, lateral epicondyle and phalanx 4 marker on each limb. We used a global angle for three markers to calculate an approximate Elbow angle. We had no access to an inverse kinematic model for dog, which would have given the best estimate. Under these circumstances the global angle was deemed the best choice as opposed to for example a dog sagittal plane calculation

A number of symmetry indexes and a plot routine for them were constructed. Matlab code below. The function can be fed with vertical impulse or peak force, either normalized or absolute values.

Abbreviations used LF = Left Fore, RF = Right Fore, LH = Left Hind and RH = Right Hind.

Symmetry indexes were calculated as follows.

1. A fore/hind symmetry index were calculated for the actual recording.
   ForeHindSymAct = (LF+RF)/(LF+RF+LH+RH)
2. Symmetry index for left and right forelimb in relation to each other.
   LeftForeQuote = - LF/(LF+RF)
   RightForeQuote = LeftForeQuote+1
3. In the same way indexes for hind limbs.
   LeftHindQuote = - LH/(LH+RH)

RightHindQuote = RH/(LH+RH)

1. Ipsilateral symmetry indexes for left and right side limbs.
   LeftIpsiQuote = LF/(LF+LH)-ForeHindSymAct;

RightIpsiQuote = RF/(RF+RH)-ForeHindSymAct;

A plot of these indexes was constructed creating a template for a sound dog, that is, a dog with evenly distributed forces between the four limbs taking into account the normal distribution of body weight between fore- and hindlimbs. In this case 11 dogs in our control group were used to calculate what is used as normal in this paper. It is the average peak vertical force quotient between fore- and hindlimbs measured under all trials with the 11 dogs and it produced the value 0.635.

The sound dog was then represented by a unit square (side length = 1) and divided into for rectangles with a centered vertical line and a horizontal line offset 0.635 down from the upper line in the square

Next, the values of the actual symmetry of dog under test were plotted by offsetting each corner exactly the relative amount calculated in the different indexes. If you have a dog that is not perfectly symmetrical in its load pattern between the four limbs this graph show a polygon that is skewed in relation to the “sound” square and graphically representing the amount and direction (forward backward and sideways) of how limbs are loaded. The very simple interpretation is that the more a corner is skewed inwards the more that limb is unloaded compared to the even situation and likewise more loaded when outside the square.

A measure of total asymmetry was created by subtracting the union of the square and the polygon from dog under test from 1. Simply put: The area that is not covered by both the square and the polygon represents the total asymmetry of the dog under test.

Total asymmetry = 1 - union(area of Test polygon, area of Normal square)

Impulse was calculated as the area under the force curve with Matlab function cumtrapz

Speed was calculated as mean displacement of all upper body markers over time

Direction was detected by horizontal force sign just after impact

Matlab code to draw the symmetry plot

function Sym = DogForcePlot(LF,RF,LH,RH,axesForcePlot,Color,ForeHindSymGuide)

% Used to plot Peak force or Impulse, either as weight normalized or

% absolute values as inputs in a symmetry plot

% Lars Roepstorff

% Swedish University of Agricultural Sciences

% Original 2020-11-16, last update 2021-11-06

if nargin == 0 % Example data if no input arg

LF = 172;

RF = 216;

LH = 132;

RH = 117;

scrsz = get(groot,'ScreenSize');

fh = figure('Position',[1 scrsz(4) scrsz(3) scrsz(4)],'NumberTitle','off');

axesForcePlot = axes('Units','pixels','Parent',fh,'FontSize',10,...

'XTick',[],'Position',[30 30 scrsz(3)-50 scrsz(4)-100]);

ForeHindSymGuide = 0.635; % Calculated fore/hind distribution, based on 11 control dogs

elseif nargin<7 % If no symmetry guide, i.e. normal dogs is specified use the value from paper

ForeHindSymGuide = 0.635;

elseif nargin<6 % Default color if nothing is specified

Color = 'r';

elseif nargin<5 % If no plot axes is defined create one

scrsz = get(groot,'ScreenSize');

fh = figure('Position',[1 scrsz(4) scrsz(3) scrsz(4)],'NumberTitle','off');

axesForcePlot = axes('Units','pixels','Parent',fh,'FontSize',10,...

'XTick',[],'Position',[30 30 scrsz(3)-50 scrsz(4)-100]);

end

% Force Symmetry Plot

ForeHindSymAct = (LF+RF)/(LF+RF+LH+RH);

LeftForeQuote = - LF/(LF+RF);

RightForeQuote = LeftForeQuote+1;

LeftHindQuote = - LH/(LH+RH);

RightHindQuote = RH/(LH+RH);

LeftIpsiQuote = LF/(LF+LH)-ForeHindSymAct;

RightIpsiQuote = RF/(RF+RH)-ForeHindSymAct;

% Plot guide

plot(axesForcePlot,[0.5 0.5 -0.5 -0.5 0.5],...

[ForeHindSymGuide-1 ForeHindSymGuide ForeHindSymGuide ForeHindSymGuide-1 ForeHindSymGuide-1],'k','LineWidth',2)

hold (axesForcePlot,'on')

plot(axesForcePlot,[0 0],[ForeHindSymGuide-1 ForeHindSymGuide],'k','LineWidth',2)

plot(axesForcePlot,[-0.5 0.5],[0 0],'k','LineWidth',2)

plot(axesForcePlot,[LeftForeQuote, RightForeQuote, RightHindQuote, LeftHindQuote, LeftForeQuote],...

[ForeHindSymAct + LeftIpsiQuote, ForeHindSymAct + RightIpsiQuote,...

ForeHindSymAct-1 + RightIpsiQuote, ForeHindSymAct-1 + LeftIpsiQuote, ...

ForeHindSymAct + LeftIpsiQuote],Color,'LineWidth',2)

% The normal or template area.

NormalPgon = polyshape([0.5 0.5 -0.5 -0.5 0.5],...

[ForeHindSymGuide-1 ForeHindSymGuide ForeHindSymGuide ForeHindSymGuide-1 ForeHindSymGuide-1]);

TestPgon = polyshape([LeftForeQuote, RightForeQuote, RightHindQuote, LeftHindQuote, LeftForeQuote],...

[ForeHindSymAct + LeftIpsiQuote, ForeHindSymAct + RightIpsiQuote,...

ForeHindSymAct-1 + RightIpsiQuote, ForeHindSymAct-1 + LeftIpsiQuote, ...

ForeHindSymAct + LeftIpsiQuote]);

TotPgon = union(TestPgon, NormalPgon);

TotArea = area(TotPgon);

Sym.TotAsym = -(TotArea-1);

Sym.ForHindSym = ForeHindSymAct-ForeHindSymGuide;

Sym.ForeSym = 0.5+LeftForeQuote;

Sym.HindSym = 0.5+LeftHindQuote;

Sym.DiagSym = LeftIpsiQuote;
